# Long-Term Persistence of Olfactory and Gustatory Disorders in COVID-19 Patients

**DOI:** 10.3389/fmed.2022.794550

**Published:** 2022-02-25

**Authors:** Nhu Ngoc Nguyen, Van Thuan Hoang, Thi Loi Dao, Line Meddeb, Sébastien Cortaredona, Jean-Christophe Lagier, Matthieu Million, Didier Raoult, Philippe Gautret

**Affiliations:** ^1^Aix Marseille University, IRD, AP-HM, SSA, VITROME, Marseille, France; ^2^IHU-Méditarranée Infection, Marseille, France; ^3^Family Medicine Department, International Relations Department, Group for Research in Emerging and Re-Emerging Infectious Diseases, Thai Binh University of Medicine and Pharmacy, Thai Binh, Vietnam; ^4^Pneumology Department, Group for Research in Emerging and Re-Emerging Infectious Diseases, Thai Binh University of Medicine and Pharmacy, Thai Binh, Vietnam; ^5^Aix Marseille University, IRD, AP-HM, MEPHI, Marseille, France

**Keywords:** SARS-CoV-2, COVID-19, smell, taste, persistence, long COVID

## Abstract

Smell and taste disorders are frequent symptoms during acute COVID-19 and may persist long after the resolution of the initial phase. This study aims to estimate the proportion and risk factors for smell and/or taste disorders at the onset of symptoms and their persistence after more than 6 months of follow-up in COVID-19 patients. We analyzed a prospective cohort of COVID-19 patients admitted to our institute in Marseille, France in early 2020. After being discharged from the hospital, patients with smell and/or taste disorders were contacted for a telephone interview. Logistic regression analysis was performed to determine the risk factors for smell and/or taste disorders. A total of 3,737 patients were included, of whom 1,676 reported smell and/or taste disorders at the onset of symptoms. Taste and/or smell disorders were independently associated with being younger and female, a lower likelihood of suffering from diabetes, cardiovascular diseases and cancer, a longer delay between the onset of symptoms and consultation, and non-severe forms of COVID-19 at admission. Of the 605 patients with smell and/or taste disorders who were followed-up, 154 (25.5%) reported the persistence of symptoms for more than 6 months. At the time of follow-up, being female, having a chronic respiratory disease and using angiotensin-converting enzyme inhibitors (ACEis) were factors independently associated with the persistence of smell and/or taste disorders. In conclusion, the long-term persistence of olfactory and gustative disorders is frequent among COVID-19 patients, notably affecting female patients and patients who suffered from chronic respiratory diseases before infection. The role of ACEis needs to be further evaluated in larger numbers of patients.

## Introduction

The clinical symptoms of COVID-19 patients vary from being asymptomatic to being fatal, with the involvement of multiple organs (1). Olfactory and gustatory disorders are frequent symptoms reported at an early phase of the disease.

In Chinese studies, the frequency of olfactory and gustatory dysfunction ranged, respectively, from 5.1 and 5.6% in one study conducted on 214 patients (2) to 39.5 and 38.4%, in another performed on 86 patients (3). One study conducted in the United Kingdom and the United States among patients with respiratory symptoms showed that loss of smell and taste was more frequent in SARS-CoV-2 positive patients than in negative patients, with a rate of 65.03% compared to 21.71% (4). A cross-sectional study conducted on 417 COVID-19 patients who were recruited from 12 European hospitals showed that 85.6 and 88.0% reported smell and taste disorders, respectively (5). In our experience, in Marseille, France, 39.2 and 37.8% of 3,737 SARS-CoV-2-infected patients seen between 3 March and 27 April reported smell and taste disorders during the acute phase, respectively (6).

In the study conducted on 417 patients in 12 European hospitals, 72.6% reported an improvement in their smell and taste functions within the first 8 days following recovery (5). In a study conducted on 588 patients in Norway, 18.0% of patients reported an altered sense of smell or taste after 8 months of follow-up (7). Recently, a large study conducted using an online survey platform in 56 countries showed that among 3,762 patients reported prevalence of changes in smell and/or taste at seven months post-onset, at 25.2% (8).

In a preliminary report, we observed that 30 out of 125 (24.0%) patients who reported smell and/or taste disorders during the initial acute phase of COVID-19 reported a persistence of these symptoms 6 months after onset, with female patients more likely to report persistent symptoms (9).

In this study, we aim to identify the proportion of persistence of olfactory and gustatory dysfunction after at least 6 months of follow-up in a larger cohort of COVID-19 patients. We also investigated risk factors for persisting symptoms.

## Materials and Methods

### Study Design and Data Collection

We conducted an observational study of PCR-confirmed COVID-19 patients (10) who reported smell and/or taste disorders during the acute phase upon admission to our institute in 2020. Information on demographics, co-morbidities, co-medications, and clinical data at inclusion and upon treatment were retrospectively retrieved from medical files and have previously been published elsewhere (6). Severity was evaluated using the National Early Warning Score for COVID-19 patients (NEWS-2) (11) with three categories: low score (NEWS-2 = 0–4), medium (NEWS-2 = 5–6) and high score (NEWS-2 ≥ 7). More than 6 months after discharge, patients were invited by telephone to complete a questionnaire to evaluate the duration of their taste and smell disorders and partial or complete recovery. Interviews were conducted by a group of 24 physicians using a standardized questionnaire (Supplementary Material).

### Data Analysis

A minimum sample size of 529 patients was calculated for a confidence level of 95%, a proportion of persistence of 24.0% (9), and a margin of error set at a 3% confidence limit (12, 25). Statistical analysis was performed using the open-source software R (R Core Team. R: A language and environment for statistical computing. R Foundation for statistical computing, Vienna, Australia, 2020. URL: http://www.r-project.org).

Data are presented as numbers and percentages for categorical variables and mean ± standard deviation (SD) for continuous variables. The Chi-square test was applied to compare the differences between proportions.

We investigated the risk factors for smell and/or taste disorders during the acute phase and risk factors for the persistence of these symptoms more than 30 weeks following the onset of COVID-19 symptoms.

Unadjusted associations were assessed between multiple factors (socio-demographic characteristics, co-morbidities and co-medications, severity score and viral load upon initial admission) and smell and taste disorders. Variables with a *p*-value < 0.2 in the univariate analysis were included in the multivariate logistic regression model to identify risk factors for smell and/or taste disorders at the acute phase or for the persistence of these symptoms. Multi-co-linearity among the independent variables was tested using the ϕ coefficient. When pairs of variables were highly correlated (absolute value of correlation coefficient > 0.7), one variable only was entered into the multivariate model. Step-by-step descending regression was used to select the final model with the weakest Akaike Information Criterion (AIC). The odds ratio (OR) presented the results with a 95% confidence interval (95% CI).

Any variable with a prevalence of missing data of more than 5% was excluded from the multivariate analysis. A *p*-value of <0.05 was considered statistically significant.

### Ethical Approval

This study was approved by the Comité de Protection des Personnes Nord Ouest II (No. 2021-A01183-33) on 22/07/2021.

## Results

### Patient Status Upon Inclusion

Of the 3,737 COVID-19 patients diagnosed within the first months of 2020, 1,676 reported smell and/or taste disorders during the acute phase of the disease (6). The baseline characteristics of the 3,737 patients during the acute phase have been described elsewhere (6). Briefly, the mean age of the patients was 45, 45% were male, 84% received at least a 3-day course of hydroxychloroquine and azithromycin (HCQ-AZ), 18% were hospitalized and the case fatality rate was 0.9%.

Patients with smell and/or taste disorders were significantly younger than those without these symptoms. They were also more likely to be female and were less likely to report chronic conditions and co-medications (Table 1). They were more likely to consult late after the onset of symptoms, less likely to suffer from severe COVID-19, to present with high viral load upon admission, and present persistent viral shedding at 10 days post-admission. In multivariate analysis, smell and/or taste disorders continued to be associated with being younger and female, lower likelihood of suffering diabetes, cardiovascular diseases and cancer, a longer delay between the onset of symptoms and consultation and non-severe forms of COVID-19 at admission. It was noted that a high viral load (Ct value <16 at admission) was negatively associated with the presence of smell and/or taste disorders at the acute phase of COVID-19 in univariate analysis. However, this variable was not included in the multivariate analysis, given the high prevalence of missing data (19.8%) (Table 1).

**Table 1:** Risk factors for smell and/or taste disorders during the acute phase (*n* = 3,737).

		**No smell and/or taste disorders** **(*n* = 2,061) %**	**Smell and/or taste disorders** **(*n* = 1,676) %**	**Univariate analysis**		Multivariate analysis^**^	
				**OR (95%CI)**	***p*-value**	**OR (95%CI)**	***p*-value**
Age	Mean ± SD	48.95 ± 18.0	40.74 ± 13.86				
	Range	18–98	18–89				
	<45 (*n* = 1,874)	41.6	60.7	Ref		Ref	
	≥45 (*n* = 1,863)	58.4	39.3	0.46 (0.40–0.53)	**<0.001**	0.54 (0.47–0.62)	**<0.001**
Sex	Male (*n* = 1,704)	48.9	41.5	Ref		Ref	
	Female (*n* = 2,033)	51.1	58.5	1.35 (1.18–1.54)	**<0.001**	1.33 (1.16–1.52)	**<0.001**
**Chronic conditions**							
Hypertension	No (*n* = 3,176)	81.4	89.4	Ref			
	Yes (*n* = 561)	18.6	10.6	0.52 (0.42–0.63)	**<0.001**		
Diabetes	No (*n* = 3,425)	89.2	94.6	Ref		Ref	
	Yes (*n* = 312)	10.8	5.4	0.47 (0.36–0.61)	**<0.001**	0.73 (0.55–0.95)	**0.02**
Chronic	No (*n* = 3,399)	90.1	92.0	Ref			
	Yes (*n* = 338)	9.9	8.0	0.79 (0.62–1.00)	**0.04**		
respiratory							
disease							
Cardiovascular	No (*n* = 3,518)	91.5	97.4	Ref		Ref	
disease							
	Yes (*n* = 219)	8.5	2.6	0.28 (0.20–0.40)	**<0.001**	0.46 (0.32–0.66)	**<0.001**
Cancer	No (*n* = 3,608)	95.1	98.3	Ref		Ref	
	Yes (*n* = 129)	4.9	1.7	0.33 (0.21–0.51)	**<0.001**	0.49 (0.31–0.77)	**0.002**
Obesity	No (*n* = 3,319)	88.0	89.8	Ref			
	Yes (*n* = 418)	12.0	10.2	0.83 (0.67–1.03)	**0.09**		
Rhinitis	No (*n* = 3,726)	99.7	99.8	Ref			
	Yes (*n* = 11)	0.3	0.2	0.70 (0.15–2.77)	0.58		
**Co-medications**							
Beta blockers	No (*n* = 3,586)	94.7	97.6	Ref			
	Yes (*n* = 151)	5.3	2.4	0.44 (0.30–0.65)	**<0.001**		
Dihydropyridine	No (*n* = 3,598)	95.6	97.1	Ref			
	Yes (*n* = 139)	4.4	2.9	0.64 (0.44–0.92)	**0.013**		
Angiotensin-	No (*n* = 3,687)	98.2	99.2	Ref			
converting							
enzyme							
inhibitors							
	Yes (*n* = 50)	1.8	0.8	0.43 (0.21–0.83)	**0.007**		
Angiotensin II	No (*n* = 3,566)	94.4	96.7	Ref			
receptor							
blocker							
	Yes (*n* = 171)	5.6	3.3	0.57 (0.40–0.80)	**0.001**		
Metformin	No (*n* = 3,609)	96.2	97.0	Ref			
	Yes (*n* = 128)	3.8	3.0	0.78 (0.53–1.14)	**0.18**		
Fenofibrate	No (*n* = 3,716)	99.3	99.6	Ref			
	Yes (*n* = 21)	0.7	0.4	0.61 (0.21–1.63)	0.29		
Statin	No (*n* = 3,597)	95.1	97.6	Ref			
	Yes (*n* = 140)	4.9	2.4	0.48 (0.32–0.70)	**<0.001**		
**COVID-19 status at inclusion**							
Time between	<6 days (*n* = 1,806)	52.9	42.7	Ref		Ref	
onset of							
COVID symptoms							
and admission							
	≥6 days (*n* = 1,931)	47.1	57.3	1.51 (1.32–1.73)	**<0.001**	1.81 (1.59–2.08)	**<0.001**
NEWS score 2	Low (*n* = 3,420)	87.9	96.0	Ref		Ref	
	Medium (*n* = 172)	6.4	23.3	0.34 (0.24–0.49)	**<0.001**	0.48 (0.33–0.70)	**<0.001**
	High (*n* = 145)	5.7	1.6	0.26 (0.17–0.39)	**<0.001**	0.37 (0.24–0.58)	**<0.001**
PCR Ct value	No (*n* = 2,808)	92.3	95.1	Ref			
< 16 at							
admission^*^^N=2,998^	Yes (*n* = 190)	7.7	4.9	0.61 (0.45–0.84)	**0.002**		
Viral shedding ≥							
10 days^*^^N=2,412^	No (*n* = 2,032)	80.6	88.3	Ref			
	Yes (*n* = 380)	19.4	11.7	0.55 (0.44–0.70)	**<0.001**		

*NEWS Score 2: National Early Warning Score*.

* *Ct <16 and viral shedding were not included in the multivariate analysis due to missing data > 5%*.

** *Only significant results are presented in the multivariate analysis*.

*Significant p-values are indicated in bold*.

When analyzing smell and taste disorders separately, very similar results were found, except for diabetes associated with taste disorder only and obesity associated with smell disorder in the multivariate analysis (Supplementary Tables 1, 2).

### Patient Status at Follow-Up

**Figure 1:**
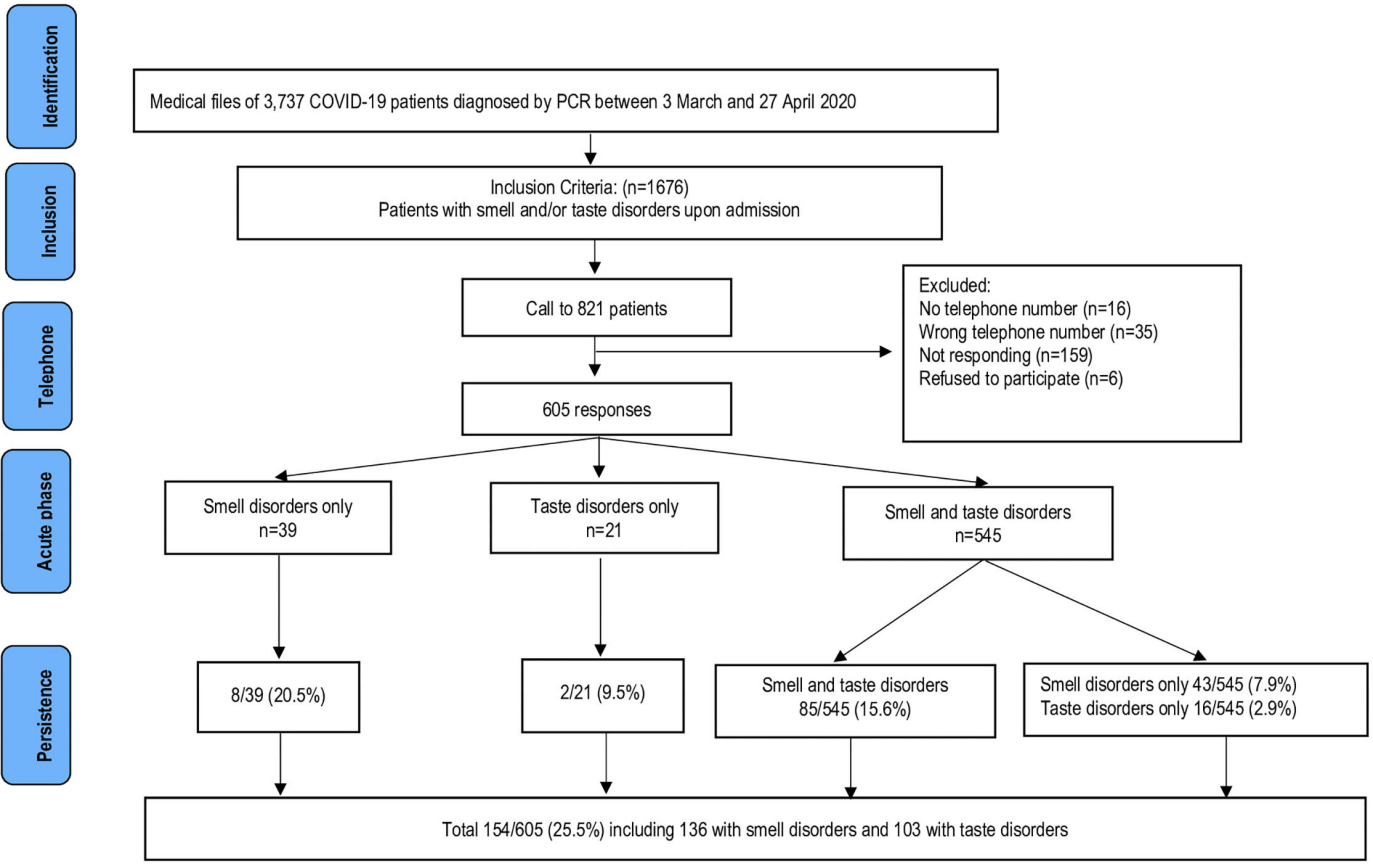
Flow-chart of selection of the study population for smell and/or taste disorders.

A total of 821 patients with smell and/or taste disorders at admission, were contacted by telephone, and 605 (73.7%) patients answered the questionnaire, including 584 patients with smell disorders and 566 patients with taste disorders (Figure 1). Most characteristics of these 605 patients did not significantly differ from those of all patients with olfactory and/or gustatory disorders during the acute phase. The proportion of female patients among the respondents was, however, significantly higher (63.8 vs. 58.5%), the proportion of patients receiving dihydropyridine was lower (1.3 vs. 2.9%), and the proportion of patients with a delay between onset of symptoms and admission ≥ 6 days was lower (54.5 vs. 57.3%) (Table 2).

**Table 2:** Characteristics of COVID-19 patients with smell and/or taste disorders during the acute phase.

		**All patients (*n* = 1,676)**	**Patients with follow-up at**	***p*-value**
			**more than 6 months (*n* = 605)**	**(chi-square test)**
Age (years)	Mean ± SD Range	40.7 ±13.9 18–89	40.0 ± 13.3 18–89	
Sex	Male	696 (41.5)	219 (36.2)	**0.022**
	Female	980 (58.5)	386 (63.8)	
**Chronic conditions**				
Hypertension		177 (10.6)	65 (10.7)	0.90
Diabetes		90 (5.4)	30 (5.0)	0.69
Chronic respiratory disease		134 (8.0)	62 (10.2)	0.09
Chronic cardiovascular disease		43 (2.6)	13 (2.1)	0.57
Cancer		28 (1.7)	12 (2.0)	0.62
Obesity		171 (10.2)	62 (10.2)	0.98
Rhinitis		4 (0.2)	1 (0.2)	0.74
**Co-medications**				
Beta blocker		41 (2.4)	15 (2.5)	0.97
Dihydropyridine		48 (2.9)	8 (1.3)	**0.036**
Angiotensin-converting enzyme inhibitor		13 (0.8)	9 (1.5)	0.13
Angiotensin II receptor blocker		55 (3.3)	12 (2.0)	0.105
Metformin		50 (3.0)	12 (2.0)	0.19
Fenofibrate		7 (0.4)	3 (0.5)	0.803
Statin		40 (2.4)	7 (1.2)	0.07
**COVID-19 status at inclusion**				
Time between onset of symptoms		961 (57.3)	330 (54.5)	**<0.001**
and admission ≥ 6 days				
NEWS Score-2	Low (NEWS-2 = 0–4)	1,609 (96.0)	588 (97.2)	0.39
	Medium (NEWS-2 = 5–6)	40 (2.4)	11 (1.8)	
	High (NEWS-2 ≥ 7)	27 (1.6)	6 (1.0)	
PCR Ct value <16 at admission^N=2,998,537^		71 (4.9)	35 (6.5)	0.15
Viral shedding ≥ 10 days^N=2,412,369^		134 (11.7)	53 (14.4)	0.19
Hydroxychloroquine + azithromycin ≥ 3 days		1,475 (88.0)	546 (90.2)	0.14

*NEWS Score 2: National Early Warning Score*.

*Significant p-values are indicated in bold*.

**Figure 2:**
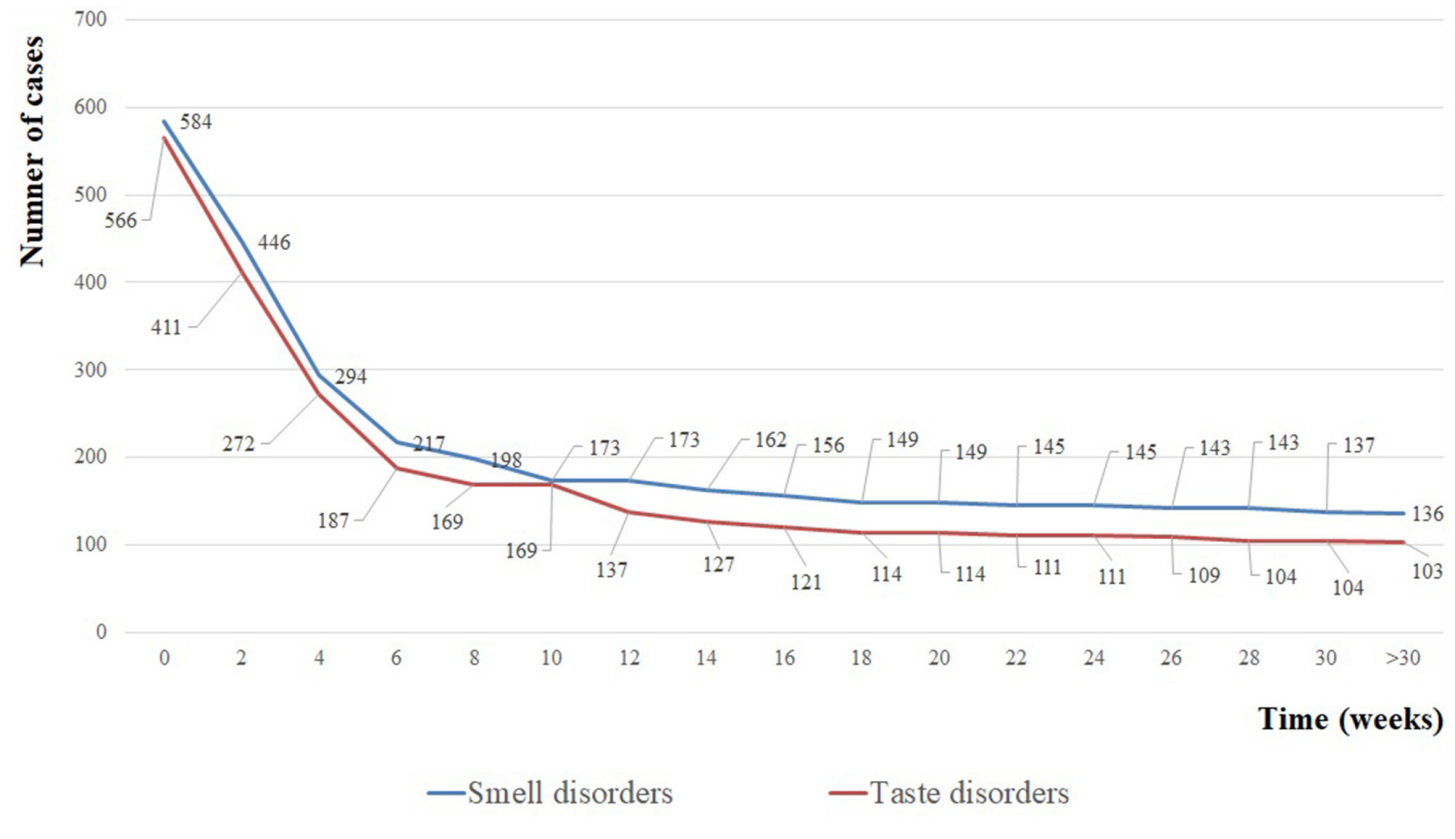
Number of patients with persistence of smell disorders (blue curve) and taste disorders (red curve) over time.

Of the 605 respondents, 545 reported smell disorders and taste disorders, while 39 only reported smell disorders and 21 only reported taste disorders during the acute phase. Figure 1 shows the persistence of symptoms according to the initial status. The number of patients with persisting symptoms rapidly dropped during the first 6 weeks following the onset of symptoms and remained relatively stable from week 10 until the end of follow-up (Figure 2). During the first month after hospital discharge, 365/448 (81.5%) and 377/463 (81.4%) patients reported smell and taste recovery, respectively. Of note, 293/365 (80.3%) and 304/377 (80.6%) reported having recovered 100% of their smell and taste function, while 72/365 (19.7%) and 73/377 (19.4%) reported a only partial recovery of smell and taste during the first month. At follow-up, 154/605 (25.5%) patients reported the persistence of smell and/or taste disorders after more than 6 months. Of these 154 patients, 136 reported the persistence of smell disorders and 103 reported the persistence of taste disorders. Of patients with persistent smell disorders, 117/136 (86.1%) had partially recovered and 19/136 (13.9%) had experienced no recovery at all. For those with taste disorders, 88/103 (85.4%) had partially recovered and 15/103 (14.6%) had experienced no recovery at all. Older age, female sex, chronic respiratory diseases and the use of ACE inhibitors (ACEis) were significantly associated with the persistence of smell and/or taste disorders at 30 weeks of follow-up, while obesity was associated with the resolution of symptoms (Table 3). In multivariate analysis, being female, having a chronic respiratory disease and using ACEis remained significantly associated with the persistence of smell and/or taste disorders (Table 3).

**Table 3:** Risk factors for smell and/or taste disorders at the time of follow-up (*n* = 605).

		**Resolution of**	**Persistence of**	**Univariate analysis**		**Multivariate analysis** ^**^	
		**smell and/or**	**smell and/or**				
		**taste disorders**	**taste disorders**				
		**(*n* = 451)**	**(*n* = 154)**				
				**OR (95%CI)**	***p*-value**	**OR (95%CI)**	***p*-value**
				**Ref = resolution**		**Ref = resolution**	
Age	Mean ± SD	39.4 ± 13.5	41.9 ± 12.4				
	Range	18–89	18–72				
	<45 (*n* = 374)	64.5	53.9	Ref			
	≥45 (*n* = 231)	35.5	46.1	1.56 (1.07–2.25)	**0.02**		
Sex	Male (*n* = 219)	38.8	28.6	Ref		Ref	
	Female (*n* = 386)	61.2	71.4	1.59 (1.07–2.36)	**0.023**	1.64 (1.09–2.46)	**0.016**
**Chronic conditions**							
Hypertension	No (*n* = 540)	89.6	88.3	Ref			
	Yes (*n* = 65)	10.4	11.7	1.14 (0.64–2.03)	0.7		
Diabetes	No (*n* = 575)	94.7	96.1	Ref			
	Yes (*n* = 30)	5.3	3.9	0.72 (0.29–1.79)	0.48		
Chronic respiratory	No (*n* = 543)	91.8	83.8	Ref		Ref	
disease							
	Yes (*n* = 62)	8.2	16.2	2.17 (1.26–3.74)	**0.005**	1.94 (1.10–3.41)	**0.021**
Asthma	No (*n* = 559)	92.3	92.9	Ref		Ref	
	Yes (*n* = 46)	7.7	7.1	1.63 (0.86–3.07)	**0.13**		
Bronchitis	No (*n* = 598)	99.1	98.1	Ref			
	Yes (*n* = 7)	0.9	1.9	2.22 (0.49–10.03)	0.3		
Chronic	No (*n* = 592)	98.0	97.4	Ref			
cardiovascular							
disease							
	Yes (*n* = 13)	2.0	2.6	1.31 (0.39–4.31)	0.7		
Cancer	No (*n* = 593)	98.4	96.8	Ref			
	Yes (*n* = 12)	1.6	3.2	2.13 (0.67–6.81)	0.203		
Obesity	No (*n* = 543)	88.2	94.2	Ref			
	Yes (*n* = 62)	11.8	5.8	0.47 (0.22–0.97)	**0.041**		
Rhinitis	No (*n* = 604)	99.8	100.0				
	Yes (*n* = 1)	0.2	0.0	-			
**Co-medications**							
Beta blocker	No (*n* = 590)	97.6	97.4	Ref			
	Yes (*n* = 15)	2.4	2.6	1.07 (0.33–3.40)	0.91		
Dihydropyridine	No (*n* = 597)	98.4	99.4	Ref			
	Yes (*n* = 8)	1.6	0.6	0.41 (0.05–3.39)	0.41		
Angiotensin-converting	No (*n* = 596)	99.3	96.1	Ref		Ref	
enzyme inhibitors							
(1 Enalapril, 4 Ramipril,							
3 Trandolapril,							
1 Zofenopril)							
	Yes (*n* = 9)	0.7	3.9	6.05 (1.49–24.5)	**0.012**	5.23 (1.22–22.36)	**0.026**
Angiotensin II receptor	No (*n* = 593)	98.1	97.6	Ref			
blocker							
	Yes (*n* = 12)	1.9	2.4	0.58 (0.13–2.68)	0.49		
Metformin	No (*n* = 593)	98.4	96.8	Ref			
	Yes (*n* = 12)	1.6	3.2	2.13 (0.67–6.81)	0.203		
Fenofibrate	No (*n* = 602)	99.8	98.7	Ref			
	Yes (*n* = 3)	0.2	1.3	5.92 (0.53–65.76)	**0.15**		
Statin	No (*n* = 598)	98.9	98.7	Ref			
	Yes (*n* = 7)	1.1	1.3	1.17 (0.23–6.11)	0.85		
**COVID-19 status at inclusion**							
Time between the	<6 days (*n* = 276)	47.0	41.6	Ref			
onset symptoms							
and admission							
	≥6 days (*n* = 329)	53.0	58.4	1.25 (0.86–1.81)	0.24		
NEWS Score-2	Low (*n* = 588)	96.7	98.7	Ref			
	Medium (*n* = 11)	2.2	0.6	0.29 (0.04–2.26)	0.24		
	High (*n* = 6)	1.1	0.6	0.57 (0.07–4.95)	0.61		
PCR Ct value	No (*n* = 502)	94.8	89.7	Ref			
< 16 at							
admission^*^^N=537^							
	Yes (*n* = 35)	5.2	10.3	2.08 (1.02–4.21)	**0.043**		
Viral shedding	No (*n* = 316)	84.5	88.5	Ref			
≥ 10 days^*^^N=369^							
	Yes (*n* = 53)	15.5	11.5	0.70 (0.36–1.39)	0.31		
Hydroxychloroquine	No (*n* = 59)	9.8	9.7	1.00 (0.89–1.12)	0.99		
+ azithromycin							
≥ 3 days							
	Yes (*n* = 546)	90.2	90.3				

*NEWS Score 2: National Early Warning Score*.

* *Ct <16 and viral shedding were not included in the multivariate due to missing data > 5%*.

** *Only significant results are presented in the multivariate analysis*.

*Significant p-values are indicated in bold*.

Given the high prevalence of missing data, viral load at admission was not included in the multivariate analysis model, but in the univariate analysis, a high viral load (Ct value <16) was associated with the persistence of symptoms. When analyzing symptoms separately, the use of ACEis was independently associated with the persistence of smell disorders, while being older was independently associated with the persistence of taste disorders (Supplementary Tables 3, 4).

## Discussion

The proportion of 39.2 and 37.8% of patients reporting smell and taste disorders during the acute phase of COVID-19 is in line with the results of a meta-analysis showing pooled proportions of olfactory and gustatory disorders of 41.0 and 38.2% among 8,438 patients (13). In our study, 90% of patients reporting taste and/or smell disorders reported an impairment of both functions. We observed that taste and smell disorders were more frequent in patients consulting more than 6 days after the onset of symptoms, suggesting that it takes a few days for the virus to provoke such symptoms. Women generally outperform men in olfactory abilities (14) and aging is associated with decreased olfactory performance (15). It is, therefore, not surprising that younger people and women were more prone to identifying smell alterations than others in our survey. Also, olfactory and gustatory disorders were associated with non-severe forms of COVID-19 and a low viral load and with the absence of risk factors for severe COVID-19, such as diabetes, cardiovascular diseases, and cancer making these symptoms a marker of favorable evolution of the disease. In contrast to our result, a relation between viral load in saliva and smell/taste alterations were observed in another work (16). In symptomatic patients, SARS-CoV-2 viral load in saliva was 5-time higher in patients reporting a loss of taste and smell (16). In fact, oral keratinocytes, and those from the taste buds are directly targeted by SARS-CoV-2. This could explain the etiopathogenesis of smell/taste alterations (16) and the longer persistence of SARS-CoV-2 in saliva as compared to the oropharynx (17). The association of symptom persistence with being older may suggest a lower cell regeneration capacity due to aging. In our series, we have no hypothesis to explain the lower capacity of olfactory and gustatory symptom resolution in women and patients suffering from chronic respiratory diseases (mostly asthma). It cannot be excluded that potential confounding factors which were not investigated, including tobacco use and alcohol consumption may have played a role. Remarkably, we observed that the strongest determinant of the persistence of smell disorder in our patients was the treatment with ACEis. This result, although statistically significant, is based on very few patients and further studies are needed to confirm this observation. Furthermore, we found no independent association between smell and taste disorders during the acute phase of the disease and the use of ACEis.

At present, there are no specific drugs for smell and/or taste disorders associated with COVID-19. Systemic and nasal corticosteroids are not recommended for COVID-19 patients with smell and/or taste disorders (5, 18). As a consequence, no specific treatment was proposed to patients with smell and taste disorders at our institute during the acute phase of the disease. Some authors have performed nasal irrigation studies (including an intranasal steroid/mucolytics/decongestant solution), however, we need more data to confirm the effectiveness of these treatments (19). The effectiveness of olfactory training was observed in post-traumatic and post-infectious patients (20, 21). In this non-pharmacological treatment, patients expose themselves twice daily to four different odors (phenyl ethyl alcohol: rose, eucalyptol: eucalyptus, citronellal: lemon, and eugenol: cloves) from 12 to 24 weeks (22). The exact underlying mechanism of improving the smell function by this method remains unclear. It is proposed that repeatedly smelling these odors increases the regenerative capacity of the smell neurons (23). One prospective cohort study was conducted on 27 COVID-19 patients with smell disorders which included nine patients treated by oral corticosteroids and olfactory training and 18 patients who performed olfactory training only. Only patients treated with a combination of oral corticosteroids and olfactory training showed a significant improvement in smell capacity after 10 weeks of follow-up (24). To confirm the effectiveness of olfactory training in COVID-19 patients, further studies with a larger sample size are needed.

We acknowledge that our study had some limitations. Firstly, this study was based on a telephone interview, which made it difficult to quantify changes in smell and taste in COVID-19 patients. Studies based on the evaluation of patients through clinical tests are needed. Secondly, some patients did not answer phone calls and were therefore lost to follow-up, which could be explained by many reasons, including a total recovery of symptoms, which could have affected the proportion of smell and taste disorders which persisted at follow-up. Thirdly, re-testing of viral load was not possible at follow-up. Fourthly, we did not query patients with persistent symptoms about olfactory training. Finally, we did not queried information about the oral condition during the acute phase.

In conclusion, the long-term persistence of olfactory and gustative disorders is frequent in COVID-19 patients, particularly affecting female patients and patients who were suffering from chronic respiratory disease before infection. The role of ACEis needs to be further evaluated in larger numbers of patients.

## Data Availability Statement

The datasets analyzed in this study will be available by request to the corresponding author. Requests to access these datasets should be directed to Dr. Philippe Gautret, philippe.gautret@club-internet.

## Ethics Statement

The studies involving human participants were reviewed and approved by the Comité de Protection des Personnes Nord Ouest II (No. 2021-A01183-33). The patients/participants provided their written informed consent to participate in this study.

## Author Contributions

NN and VH: conceptualization, investigation, formal analysis, review, and editing. TD and SC: formal analysis, review, and editing. LM, J-CL, and MM: investigation, review, and editing. DR: conceptualization, review, and editing. PG: conceptualization, investigation, formal analysis, writing the original draft, review, and editing. All authors have contributed to this study and approved the final version of manuscript.

## Funding

This research was supported by the Fondation Méditerranée Infection.

## Conflict of Interest

The authors declare that the research was conducted in the absence of any commercial or financial relationships that could be construed as a potential conflict of interest.

## Publisher's Note

All claims expressed in this article are solely those of the authors and do not necessarily represent those of their affiliated organizations, or those of the publisher, the editors and the reviewers. Any product that may be evaluated in this article, or claim that may be made by its manufacturer, is not guaranteed or endorsed by the publisher.
