# Supplementary material for: Long-Term Persistence of Olfactory and Gustatory Disorders in COVID-19 Patients
Source: Front Med (Lausanne). 2022 Feb 25;9:794550. doi: 10.3389/fmed.2022.794550 (PMC8915119; doi:10.3389/fmed.2022.794550)
Supplement: Supplementary file 1 [file Data_Sheet_1.docx]

**Supplementary Table 1.** Risk factors for smell disorders at acute phase (n=3737)

|  |  | **No smell disorders**  **(n=2286)**  **%** | **Smell disorders**  **(n=1451)**  **%** | **Univariate analysis** | **Multivariate analysis**** |
| --- | --- | --- | --- | --- | --- |
|  |  |  |  | **OR**  **[95%CI]**  **p-value** | **OR**  **[95%CI]**  **p-value** |
| Age | Mean ± SD | 48.7 ± 17.7 | 39.9 ± 13.6 |  | |
|  | Range | 18-98 | 18-89 |  | |
|  | < 45 (n=1874) | 42.0 | 62.9 | ref | ref |
|  | ≥ 45 (n=1863) | 58.0 | 37.1 | 0.43  [0.37-0.49]  **<0.0001** | 0.48  [0.42-0.56]  **<0.0001** |
| Sex | Male (n=1704) | 48.6 | 40.9 | ref | ref |
|  | Female (n=2033) | 51.4 | 59.1 | 1.37  [1.20-1.57]  **<0.0001** | 1.36  [1.18-1.56]  **<0.0001** |
| **Chronic conditions** | | | | | |
| Hypertension | No (n=3176) | 81.7 | 90.2 | ref |  |
|  | Yes (n=561) | 18.3 | 9.8 | 0.48  [0.39-0.59]  **<0.0001** |  |
| Diabetes | No (n=3425) | 89.6 | 94.8 | ref |  |
|  | Yes (n=312) | 10.4 | 5.2 | 0.47  [0.36-0.62]  **<0.0001** |  |
| Chronic respiratory disease | No (n=3399) | 90.5 | 91.7 | ref |  |
|  | Yes (n=338) | 9.5 | 8.3 | 0.86  [0.67-1.09]  **0.19** |  |
| Cardiovascular disease | No (n=3518) | 92.0 | 97.5 | ref | ref |
|  | Yes (n=219) | 8.0 | 2.5 | 0.30  [0.21-0.44]  **<0.0001** | 0.49  [0.34 – 0.72]  **<0.0001** |
| Cancer | No (n=3608) | 95.5 | 98.2 | ref | ref |
|  | Yes (n=129) | 4.5 | 1.8 | 0.39  [0.24-0.60]  **<0.0001** | 0.59  [0.37 – 0.93]  **0.03** |
| Obesity | No (n=3319) | 87.8 | 90.4 | ref | ref |
|  | Yes (n=418) | 12.2 | 9.6 | 0.77  [0.62-0.96]  **0.02** | 0.79  [0.64 – 0.99]  **0.04** |
| Rhinitis | No (n=3726) | 99.7 | 99.7 | ref |  |
|  | Yes (n=11) | 0.3 | 0.3 | 0.90  [0.19-3.55]  0.87 |  |
| **Co-medications** | | | | | |
| Beta blockers | No (n=3586) | 94.8 | 97.8 | ref |  |
|  | Yes (n=151) | 5.2 | 2.2 | 0.41  [0.27-0.62]  **<0.0001** |  |
| Dihydropyridine | No (n=3598) | 95.5 | 97.5 | ref |  |
|  | Yes(n=139) | 4.5 | 2.5 | 0.54  [0.36-0.80]  **0.001** |  |
| Angiotensin-converting enzyme inhibitors | No (n=3687) | 98.3 | 99.2 | ref |  |
|  | Yes (n=50) | 1.7 | 0.8 | 0.49  [0.23-0.97]  **0.03** |  |
| Angiotensin II receptor blocker | No (n=3566) | 94.4 | 97.0 | ref |  |
|  | Yes (n=171) | 5.6 | 3.0 | 0.51  [0.35-0.74]  **0.0002** |  |
| Metformin | No (n=3609) | 96.1 | 97.3 | ref |  |
|  | Yes (n=128) | 3.9 | 2.7 | 0.68  [0.45-1.01]  **0.05** |  |
| Fenofibrate | No (=3716) | 99.4 | 99.5 | ref |  |
|  | Yes (n=21) | 0.6 | 0.5 | 0.79  [0.27-2.09]  0.60 |  |
| Statin | No (n=3597) | 95.1 | 98.0 | ref |  |
|  | Yes (n=140) | 4.9 | 2.0 | 0.40  [0.25-0.61]  **<0.0001** |  |
| **COVID-19 status at inclusion** | | | | | |
| Time between onset of COVID symptoms and admission | < 6 days (=1806) | 51.9 | 42.7 | ref | ref |
|  | ≥ 6 days (n=1931) | 48.1 | 57.3 | 1.45  [1.26-1.65]  **<0.0001** | 1.73  [1.50-1.98]  **<0.0001** |
| NEWS Score 2 | Low (n=3420) | 88.4 | 96.4 | ref | ref |
|  | Medium (n=172) | 6.3 | 2.0 | 0.29  [0.20-0.44]  **<0.0001** | 0.41  [0.27 – 0.62]  **<0.0001** |
|  | High (n=145) | 5.3 | 1.6 | 0.27  [0.17-0.43]  **<0.0001** | 0.40  [0.25 – 0.64]  **<0.0001** |
| PCR Ct value < 16 at admission*^N=2998^ | No (n=2808) | 92.5 | 95.2 | ref |  |
|  | Yes (n=190) | 7.5 | 4.8 | 0.62  [0.44-0.86]  **0.003** |  |
| Viral shedding ≥ 10 days*^N=2142^ | No (n=2032) | 81.2 | 88.6 | ref |  |
|  | Yes (n=380) | 18.8 | 11.4 | 0.55  [0.43-0.71]  **<0.0001** |  |

NEWS Score 2: National Early Warning Score

*Ct < 16 and viral shedding were not included in the multivariate analysis due to missing data > 5%

**only significant results are presented in the multivariate analysis

**Supplementary Table 2.** Risk factor for taste disorders at acute phase (n=3737)

|  |  | **No taste disorders**  **(n=2341)**  **%** | **Taste disorders**  **(n=1396)**  **%** | **Univariate analysis** | **Multivariate analysis**** |
| --- | --- | --- | --- | --- | --- |
|  |  |  |  | **OR**  **[95%CI]**  **p-value** | **OR**  **[95%CI]**  **p-value** |
| Age | Mean ± SD | 47.8 ± 17.8 | 41.1 ± 13.9 |  | |
|  | Range | 18-98 | 18-89 |  | |
|  | < 45 (n=1874) | 44.3 | 60.0 | ref | ref |
|  | ≥ 45 (n=1863) | 55.7 | 40.0 | 0.53  [0.46-0.61]  **<0.0001** | 0.62  [0.53-0.71]  **<0.0001** |
| Sex | Male (n=1704) | 49.0 | 39.9 | ref | ref |
|  | Female (n=2033) | 51.0 | 60.1 | 1.45  [1.26-1.66]  **<0.0001** | 1.43  [1.25-1.64]  **<0.0001** |
| **Chronic conditions** | | | | | |
| Hypertension | No (n=3176) | 82.5 | 89.2 | ref |  |
|  | Yes (n=561) | 17.5 | 10.8 | 0.57  [0.46-0.70]  **<0.0001** |  |
| Diabetes | No (n=3425) | 89.8 | 94.8 | ref | ref |
|  | Yes (n=312) | 10.2 | 5.2 | 0.48  [0.36-0.63]  **<0.0001** | 0.68  [0.51–0.91]  **0.01** |
| Chronic respiratory disease | No (n=3399) | 90.4 | 92.0 | ref |  |
|  | Yes (n=338) | 9.6 | 8.0 | 0.82  [0.64-1.04]  **0.09** |  |
| Cardiovascular disease | No (n=3518) | 92.2 | 97.4 | ref | ref |
|  | Yes (n=219) | 7.8 | 2.6 | 0.32  [0.22-0.47]  **<0.0001** | 0.50  [0.34–0.73]  **<0.0001** |
| Cancer | No (n=3608) | 95.5 | 98.3 | ref | ref |
|  | Yes (n=129) | 4.5 | 1.7 | 0.37  [0.23-0.59]  **<0.0001** | 0.52  [0.32–0.83]  **0.01** |
| Obesity | No (n=3319) | 88.5 | 89.3 | ref |  |
|  | Yes (n=418) | 11.5 | 10.7 | 0.92  [0.74-1.14]  0.44 |  |
| Rhinitis | No (n=3726) | 99.7 | 99.7 | ref |  |
|  | Yes (n=11) | 0.3 | 0.3 | 0.96  [0.21-3.78]  0.95 |  |
| **Co-medications** | | | | | |
| Beta blockers | No (n=3586) | 95.1 | 97.4 | ref |  |
|  | Yes (n=151) | 4.9 | 2.6 | 0.51  [0.34-0.76]  **0.001** |  |
| Dihydropyridine | No (n=3598) | 95.7 | 97.2 | ref |  |
|  | Yes(n=139) | 4.3 | 2.8 | 0.64  [0.43-0.95]  **0.02** |  |
| Angiotensin-converting enzyme inhibitors | No (n=3687) | 98.4 | 99.1 | ref |  |
|  | Yes (n=50) | 1.6 | 0.9 | 0.53  [0.25-1.03]  **0.05** |  |
| Angiotensin II receptor blocker | No (n=3566) | 94.6 | 96.9 | ref |  |
|  | Yes (n=171) | 5.4 | 3.1 | 0.57  [0.39-0.81]  **0.001** |  |
| Metformin | No (n=3609) | 96.3 | 97.1 | ref |  |
|  | Yes (n=128) | 3.7 | 2.9 | 0.78  [0.52-1.16]  0.21 |  |
| Fenofibrate | No (=3716) | 99.4 | 99.5 | ref |  |
|  | Yes (n=21) | 0.6 | 0.5 | 0.84  [0.29-2.22]  0.70 |  |
| Statin | No (n=3597) | 95.7 | 97.2 | ref |  |
|  | Yes (n=140) | 4.3 | 2.8 | 0.64  [0.43-0.94]  **0.02** |  |
| **COVID-19 status at inclusion** | | | | | |
| Time between onset of COVID symptoms and admission | < 6 days (=1806) | 52.0 | 42.2 | ref | ref |
|  | ≥ 6 days (n=1931) | 48.0 | 57.8 | 1.48  [1.29-1.70]  **<0.0001** | 1.72  [1.50-1.98]  **<0.0001** |
| NEWS Score 2 | Low (n=3420) | 89.0 | 95.7 | ref | ref |
|  | Medium (n=172) | 5.8 | 2.6 | 0.41  [0.28-0.60]  **<0.0001** | 0.57  [0.38–0.83]  **0.004** |
|  | High (n=145) | 5.2 | 1.7 | 0.31  [0.20-0.48]  **<0.0001** | 0.43  [0.27–0.69]  **<0.001** |
| PCR Ct value < 16 at admission* ^N=2998^ | No (n=2808) | 92.8 | 94.9 | ref |  |
|  | Yes (n=190) | 7.2 | 5.1 | 0.69  [0.50-0.96]  **0.02** |  |
| Viral shedding ≥ 10 days* ^N=2412^ | No (n=2032) | 81.5 | 88.5 | ref |  |
|  | Yes (n=380) | 18.5 | 11.5 | 0.57  [0.45-0.73]  **<0.0001** |  |

NEWS Score 2: National Early Warning Score

*Ct < 16 and viral shedding were not included in the multivariate analysis due to missing data > 5%

**only significant results are presented in the multivariate analysis

**Supplementary Table 3.** Risk factors for smell disorders at the time of follow-up (n=584)

|  |  | **Resolution of smell disorders**  **(n=448)** | **Persistence of smell disorders**  **(n=136)** | **Univariate analysis** | **Multivariate analysis** |
| --- | --- | --- | --- | --- | --- |
|  |  |  |  | **OR**  **[95%CI]**  **p-value** | **OR**  **[95%CI]**  **p-value** |
|  |  |  |  | **ref=resolution** | **ref=resolution** |
| Age | Mean ± SD | 39.4 ± 13.3 | 41.8 ± 12.4 |  |  |
|  | Range | 18 - 89 | 18 - 72 |  |  |
|  | < 45 (n=364) | 64.3 | 55.9 | ref |  |
|  | ≥ 45 (n=220) | 35.7 | 44.1 | 1.42  [0.94-2.14]  **0.08** |  |
| Sex | Male (n=209) | 37.5 | 30.1 | ref |  |
|  | Females (n=375) | 62.5 | 69.9 | 1.39  [0.91-2.16]  **0.12** |  |
| **Chronic conditions** | | | | | |
| Hypertension | No (n=523) | 90.0 | 88.2 | ref |  |
|  | Yes (n=61) | 10.0 | 11.8 | 1.19  [0.61-2.25]  0.57 |  |
| Diabetes | No (n=544) | 94.4 | 96.3 | ref |  |
|  | Yes (n=30) | 5.6 | 3.7 | 0.65  [0.19-1.77]  0.38 |  |
| Chronic respiratory disease | No (n=522) | 90.6 | 85.3 | ref |  |
|  | Yes (n=62) | 9.4 | 14.7 | 1.67  [0.89-3.03]  **0.08** |  |
| Asthma | No (n=538) | 92.6 | 90.4 | ref |  |
|  | Yes (n=46) | 7.4 | 9.6 | 1.33  [0.62-2.69]  **0.41** |  |
| Bronchitis | No (n=577) | 98.9 | 98.5 | ref |  |
|  | Yes (n=7) | 1.1 | 1.5 | 1.32  [0.12-8.19]  0.74 |  |
| Chronic cardiovascular disease | No (n=572) | 98.2 | 97.1 | ref |  |
|  | Yes (n=12) | 1.8 | 2.9 | 1.67  [0.36-6.33]  0.41 |  |
| Cancer | No (n=573) | 98.4 | 97.1 | ref |  |
|  | Yes (n=11) | 1.6 | 2.9 | 1.91  [0.40-7.64]  0.30 |  |
| Obesity | No (n=524) | 88.4 | 94.1 | ref |  |
|  | Yes (n=60) | 11.6 | 5.9 | 0.48  [0.19-1.05]  **0.05** |  |
| Rhinitis | No (n=583) | 99.0 | 100.0 |  |  |
|  | Yes (n=1) | 0.2 | 0.0 | - |  |
| **Co-medications** | | | | | |
| Beta blocker | No (n=571) | 97.8 | 97.8 | ref |  |
|  | Yes (n=13) | 2.2 | 2.2 | 0.99  [0.17-3.91]  0.99 |  |
| Dihydropyridine | No (n=577) | 98.7 | 99.3 | ref |  |
|  | Yes(n=7) | 1.3 | 0.7 | 0.55  [0.01-4.56]  0.57 |  |
| Angiotensin-converting enzyme inhibitors  (1 Enalapril, 4 Ramipril, 3 Trandolapril, 1 Zofenopril) | No (n=575) | 99.1 | 96.3 | ref | ref |
|  | Yes (n=9) | 0.9 | 3.7 | 4.24  [0.89-21.60]  **0.02** | 4.24  [1.12-16.01]  **0.03** |
| Angiotensin II receptor blocker | No (n=573) | 98.0 | 98.5 | ref |  |
|  | Yes (n=11) | 2.0 | 1.5 | 0.73  [0.08-3.58]  0.69 |  |
| Metformin | No (n=572) | 98.2 | 97.1 | ref |  |
|  | Yes (n=12) | 1.8 | 2.9 | 1.67  [0.36-6.33]  0.41 |  |
| Fenofibrate | No (n=581) | 99.6 | 99.3 | ref |  |
|  | Yes (n=3) | 0.4 | 0.7 | 1.65  [0.03-31.93]  0.68 |  |
| Statin | No (n=577) | 98.9 | 98.5 | ref |  |
|  | Yes (n=7) | 1.1 | 1.5 | 1.32  [0.12-8.18]  0.74 |  |
| **COVID-19 status at inclusion** | | | | | |
| Time onset symptoms and admission | < 6 days(n=284) | 47.3 | 41.2 | ref |  |
|  | ≥ 6 days (n=316) | 52.7 | 58.8 | 1.28  [0.86-1.94]  0.21 |  |
| NEWS Score-2 | Low (n=569) | 97.1 | 98.4 | ref |  |
|  | Medium (n=10) | 2.0 | 0.7 | 0.36  [0.05-2.87]  0.34 |  |
|  | High (n=5) | 0.9 | 0.7 | 0.81  [0.09-7.32]  0.85 |  |
| PCR Ct value < 16 at admission* ^N=517^ | No (n=483) | 94.7 | 89.3 | ref |  |
|  | Yes (n=34) | 5.3 | 10.7 | 2.15  [0.95-4.66]  **0.04** |  |
| Viral shedding ≥ 10 days(*) ^N=362^ | No (n=309) | 84.6 | 87.6 | ref |  |
|  | Yes (n= 53) | 15.4 | 12.4 | 0.78  [0.34-1.63]  0.48 |  |
| Hydroxychloroquine + azithromycin ≥ 3 days | No (n=57) | 9.6 | 10.3 | ref |  |
|  | Yes (n=527) | 90.4 | 89.7 | 0.93  [0.48-1.89]  0.81 |  |

NEWS Score 2: National Early Warning Score

*Ct < 16 and viral sheeding were not included in the multivariate analysis because of missing data > 5%

**only significant results are presented in multivariate analysis

**Supplementary Table 4.** Risk factors for taste disorders at the time of follow-up (n=566)

|  |  | **Resolution of taste disorders**  **(n=463)** | **Persistence of taste disorders**  **(n=103)** | **Univariate analysis** | **Multivariate analysis** |
| --- | --- | --- | --- | --- | --- |
|  |  |  |  | **OR**  **[95%CI]**  **p-value** | **OR**  **[95%CI]**  **p-value** |
|  |  |  |  | **ref=resolution** | **ref=resolution** |
| Age | Mean ± SD | 39.5 ± 13.4 | 42.8 ± 12.7 |  |  |
|  | Range | 18 - 89 | 18 - 72 |  |  |
|  | < 45 (n=345) | 63.3 | 50.5 | ref | ref |
|  | ≥ 45 (n=221) | 36.7 | 49.5 | 1.69  [1.07-2.66]  **0.02** | 1.69  [1.09-2.59]  **<0.0001** |
| Sex | Male (n=203) | 36.7 | 32.0 | ref |  |
|  | Females (n=306) | 63.3 | 68.0 | 1.23  [0.77-2.01]  0.37 |  |
| **Chronic conditions** | | | | | |
| Hypertension | No (n=505) | 89.6 | 87.4 | ref |  |
|  | Yes (n=61) | 10.4 | 12.6 | 1.25  [0.59-2.46]  0.51 |  |
| Diabetes | No (n=541) | 95.0 | 98.1 | ref |  |
|  | Yes (n=25) | 5.0 | 1.9 | 0.38  [0.04-1.58]  **0.18** |  |
| Chronic respiratory disease | No (n=508) | 90.7 | 85.4 | ref |  |
|  | Yes (n=58) | 9.3 | 14.6 | 1.66  [0.82-3.22]  **0.11** |  |
| Asthma | No (n=524) | 92.9 | 91.3 | ref |  |
|  | Yes (n=42) | 7.1 | 8.7 | 1.25  [0.51-2.78]  0.57 |  |
| Bronchitis | No (n=559) | 98.9 | 98.1 | ref |  |
|  | Yes (n=7) | 1.1 | 1.9 | 1.17  [0.09-8.26]  0.86 |  |
| Chronic cardiovascular disease | No (n=554) | 97.8 | 98.1 | ref |  |
|  | Yes (n=12) | 2.2 | 1.9 | 0.89  [0.09-4.30]  0.89 |  |
| Cancer | No (n=554) | 98.1 | 97.1 | ref |  |
|  | Yes (n=12) | 1.9 | 2.9 | 1.51  [0.26-6.21]  0.54 |  |
| Obesity | No (n=507) | 89.0 | 92.2 | ref |  |
|  | Yes (n=59) | 11.0 | 7.8 | 0.68  [0.27-1.51]  0.33 |  |
| Rhinitis | No (n=565) | 99.8 | 100.0 |  |  |
|  | Yes (n=1) | 0.2 | 0.0 | - |  |
| **Co-medications** | | | | | |
| Beta blocker | No (n=551) | 97.2 | 98.1 | ref |  |
|  | Yes (n=15) | 2.8 | 1.9 | 0.69  [0.07-3.10]  0.62 |  |
| Dihydropyridine | No (n=558) | 98.5 | 99.0 | ref |  |
|  | Yes(n=8) | 1.5 | 1.0 | 0.64  [0.01-5.06]  0.67 |  |
| Angiotensin-converting enzyme inhibitors  (1 Enalapril, 4 Ramipril, 3 Trandolapril, 1 Zofenopril) | No (n=557) | 98.9 | 96.1 | ref |  |
|  | Yes (n=9) | 1.1 | 3.9 | 3.7  [0.72-17.5]  **0.04** |  |
| Angiotensin II receptor blocker | No (n=555) | 98.1 | 98.1 | ref |  |
|  | Yes (n=11) | 1.9 | 1.9 | 0.99  [0.10-4.93]  0.99 |  |
| Metformin | No (n=556) | 98.3 | 98.1 | ref |  |
|  | Yes (n=10) | 1.7 | 1.9 | 1.13  [0.11-5.76]  0.88 |  |
| Fenofibrate | No (n=563) | 99.6 | 99.0 | ref |  |
|  | Yes (n=3) | 0.4 | 1.0 | 2.26  [0.04-43.72]  0.49 |  |
| Statin | No (n=559) | 98.5 | 100.0 | ref |  |
|  | Yes (n=7) | 1.5 | 0.0 | 0.00  [0.00-2.45]  0.21 |  |
| **COVID-19 status at inclusion** | | | | | |
| Time onset symptoms and admission | < 6 days(n=257) | 46.4 | 40.8 | ref |  |
|  | ≥ 6 days (n=309) | 53.6 | 59.2 | 1.26  [0.79-1.99]  0.29 |  |
| NEWS Score-2 | Low (n=551) | 97.2 | 98.0 | ref |  |
|  | Medium (n=10) | 1.9 | 1.0 | 0.50  [0.06-3.95]  0.51 |  |
|  | High (n=5) | 0.9 | 1.0 | 1.11  [0.12-10.07]  0.92 |  |
| Ct D0 < 16* ^N=503^ | No (n=469) | 94.7 | 86.5 | ref |  |
|  | Yes (n=34) | 5.3 | 13.5 | 2.78  [1.19-6.14]  **0.005** |  |
| Viral shedding ≥ 10 days* ^N=343^ | No (n=293) | 84.5 | 88.9 | ref |  |
|  | Yes (n=50) | 15.5 | 11.1 | 0.68  [0.26-1.57]  0.35 |  |
| Hydroxychloroquine + azithromycin ≥ 3 days | No (n=57) | 10.4 | 8.7 | ref |  |
|  | Yes (n=509) | 89.6 | 91.3 | 1.21  [0.56-2.89]  0.62 |  |

NEWS Score 2: National Early Warning Score

*Ct < 16 and viral shedding were not included in the multivariate analysis because of missing data > 5%

**only significant results are presented in multivariate analysis

**Supplementary document**

Questionnaire persistance anosmie-agueusie

IDENTIFIANT (IPP):

Nom :

Prénom :

Date de naissance :

Num séjour :

Num Tel :

Date de prise en charge à l’IHU :

1-Avez-vous bien présenté une anosmie (perte de l’odorat) et/ou une agueusie (perte du goût) au cours de votre épisode initial de COVID ?

Anosmie : oui 🞎 non 🞎 Ne sait pas 🞎

Agueusie : oui 🞎 non 🞎 Ne sait pas 🞎

Si non : passer directement à question 5

2-Présentez-vous encore ces symptômes aujourd’hui

Anosmie : oui 🞎 non 🞎 Ne sait pas 🞎

Agueusie : oui 🞎 non 🞎 Ne sait pas 🞎

Si réponse négative (disparition des symptômes) :

3- Environ combien de temps ces symptômes ont-il persisté avant de disparaître ? (réponse possible en jours, semaines ou mois)

Anosmie : ……………………………………. Ne sait pas 🞎

Agueusie ……………………………………. Ne sait pas 🞎

Si réponse positive (persistance des symptômes) :

4- Aujourd’hui quel pourcentage de vos sens estimez vous avoir par rapport à votre situation avant la maladie ?

Anosmie : ……………………………………. Ne sait pas 🞎

Agueusie ……………………………………. Ne sait pas 🞎

5/ Avez-vous bien présenté les symptomes suivants:

|  | **Au cours de votre épisode initial de COVID?** | **Présenter vous ce symptome aujourd’hui?** |
| --- | --- | --- |
| Un essoufllement | oui 🞎 non 🞎 | oui 🞎 non 🞎 |
| Une fatigue | oui 🞎 non 🞎 | oui 🞎 non 🞎 |
| Des problèmes de sommeil | oui 🞎 non 🞎 | oui 🞎 non 🞎 |
| Des problèmes de concentration | oui 🞎 non 🞎 | oui 🞎 non 🞎 |
| D’autres problèmes |  |  |

S’il y a persistance d’un symptôme informer la personne qu’elle sera contactée pour lui proposer un rendez-vous afin de mieux évaluer sa situation et afin de lui proposer une prise en charge adaptée.

Le patient est-il d’accord ? Souhaite-t-il être rappelé ?

Oui 🞎 Non 🞎 Ne sait pas 🞎

Date du (des) appel(s) 1 : Identité médecin volontaire :

Date du (des) appel(s) 2 : Identité médecin volontaire :

Date du (des) appel(s) 3 : Identité médecin volontaire :

PERDU DE VUE (au moins trois appels sans succès/ ou pas de rappel du patient) : 🞎
